# Design and Evaluation of a Broadly Multivalent Adhesins-Based Multi-Epitope Fusion Antigen Vaccine Against Enterotoxigenic *Escherichia coli* Infection

**DOI:** 10.3390/vaccines13101057

**Published:** 2025-10-16

**Authors:** Yanyan Jia, Ke Yang, Qijuan Sun, Weiqi Guo, Zhihao Yang, Zihan Duan, Shiqu Zhang, Rongxian Guo, Ke Ding, Chengshui Liao, Shaohui Wang

**Affiliations:** 1 Laboratory of Functional Microbiology and Animal Health, College of Animal Science and Technology, Henan University of Science and Technology, Luoyang 471023, China; jiayanyan0120@163.com (Y.J.); 15515625937@163.com (K.Y.); 17716325075@163.com (Z.Y.);; 2 Shanghai Veterinary Research Institute, Chinese Academy of Agricultural Sciences, Shanghai 200241, China; gwq981124@163.com; 3 Ministry of Education Key Laboratory for Animal Pathogens and Biosafety, Zhengzhou 450000, China

**Keywords:** enterotoxigenic *Escherichia coli*, fimbriae, multi-epitope fusion antigen, multivalent vaccine, immune efficacy

## Abstract

**Background**: Enterotoxigenic *Escherichia coli* (ETEC) is a zoonotic pathogen causing diarrhea and mortality in infants and livestock. Its numerous serotypes necessitate the urgent development of multivalent vaccines for effective prevention, thereby reducing public health and economic threats. **Methods**: Computational bioinformatics analyses were conducted on five major ETEC adhesins structural subunits (FaeG, FanC, FasA, FimF41a, and FedF). Dominant epitopes were selected and concatenated via flexible linkers, incorporating the PADRE sequence and LTb adjuvant to design a multi-epitope fusion antigen (MEFA). The recombinant MEFA protein was expressed in a prokaryotic system. Furthermore, molecular dynamics simulations, docking, and immune simulations assessed structural stability and immunogenicity. Immunoreactivity was tested by Western blot. Murine immunization evaluated antibody responses, lymphocyte proliferation, cytokine secretion, and protection against ETEC challenge. **Results**: Structural modeling showed an extended conformation, with docking and simulations indicating strong immune activation. Western blot confirmed MEFA immunoreactivity. MEFA induced high antigen-specific antibody titers, enhanced splenocyte proliferation, and increased IFN-γ and IL-4 secretion, indicating a Th2-biased response in mice. Vaccinated mice survived lethal ETEC challenge and maintained intestinal integrity. **Conclusions**: The MEFA candidate vaccine effectively induces robust humoral and cellular immune responses and provides protection against ETEC infection, representing a promising strategy for next-generation multivalent ETEC vaccines.

## 1. Introduction

Enterotoxigenic *Escherichia coli* (ETEC) is a zoonotic pathogen that affects infants, lambs, calves, and piglets, among others. By colonizing the small intestine through adhesins and secreting enterotoxins during replication, it causes diarrhea, dehydration, and emaciation, which can be fatal in severe cases and impose substantial economic burdens on animal production [1,2]. The high pathogenicity and virulence of ETEC are mainly attributed to two key virulence factors: adhesins, which mediate adherence to small intestinal epithelial cells, and enterotoxins, which induce fluid secretion [3,4]. Predominant ETEC strains express heat-labile (LT) or heat-stable (ST) enterotoxins, along with key fimbrial adhesins such as F4 (K88), F5 (K99), F6 (987P), F18, and F41 [5]. Furthermore, fimbrial proteins play an essential role in ETEC pathogenicity by mediating host colonization [6].

The LT (heat-labile) enterotoxin typically consists of two subunits, A and B. The B subunit primarily mediates adhesion and exhibits minimal toxicity, while also possessing immunoadjuvant properties [7,8]. To reduce LT’s inherent toxicity while retaining its adjuvant activity, multiple attenuated LT mutants have been engineered, such as LTK63 and dmLT, which have been widely studied in preclinical and clinical settings [9,10]. Emerging evidence demonstrates that LTB exerts its adjuvant activity by promoting dendritic cell (DC) turnover in the spleen and augmenting their antigen-presenting capacity, thereby enhancing T cell priming efficiency [11,12]. LTB further orchestrates ganglioside-dependent immune synapse formation by inducing sustained B cell and T cell clustering and cell cycle arrest in T lymphocytes following either: (1) antigen endocytosis via GM1-rich membrane microdomains, or (2) B cell receptor (BCR)-mediated antigen internalization [13].

Antibiotics remain the primary therapeutic approach for ETEC infections in clinical practice. Nevertheless, the extensive use of antibiotics has contributed to the emergence of drug-resistant bacterial strains, significantly diminishing treatment effectiveness [14,15]. The enforcement of China’s nationwide antibiotic restriction policy since 2020 has led to recurrent outbreaks of colibacillosis in livestock, highlighting the essential role of vaccination in ETEC prevention and control. The substantial serotype diversity of ETEC results in minimal cross-protective immunity. Existing commercial vaccines (primarily mono- or bivalent formulations) show restricted efficacy against non-vaccine serotypes and demonstrate variable performance across regions, underscoring the critical demand for developing a broadly protective multivalent vaccine candidate [4]. ETEC colonization factors (K88, K99, 987P, F41, and F18) demonstrate potent immunogenic properties, making them crucial antigenic candidates for vaccine design against ETEC infection [16,17].

The antigenic epitope, defined as the minimal structural unit of an antigen, is specifically recognized by immune receptors such as B cell receptors (BCRs) and T cell receptors (TCRs), and is capable of initiating an adaptive immune response [18]. In this context, multi-epitope vaccines have emerged as a promising strategy, offering significant potential for both prophylactic and therapeutic applications against infectious diseases [19]. Rational vaccine design employing in silico approaches demonstrates significant potential to elicit durable cross-protective immunity while precisely modulating immune response intensity, and also could improve side effects to ensure the safety of vaccines [20]. To date, 102 multi-epitope vaccines targeting diverse diseases are in clinical development, including 10 candidates specifically developed against COVID-19 [18].

In this study, we analyzed FaeG (K88), FanC (K99), FasA (987P), FimF41a (F41), and FedF (F18) to identify multiple gene fragments containing dominant protective epitopes via an in silico approach [21]. The dominant B cell epitopes from the five proteins were connected using flexible linkers, and the universal T-helper (Th) cell epitopes PADRE and LT were incorporated, which were then tandemly combined to construct a novel multi-epitope fusion antigen (MEFA) LTb-PADRE-K88-K99-987P-F41-F18. First, MEFA was computationally evaluated using molecular dynamics simulations, molecular docking, and immunological modeling. Subsequently, MEFA fusion protein was expressed in a prokaryotic system and administered to BALB/c mice via immunization. The immunogenicity of this multivalent epitope vaccine was systematically evaluated by assessing both humoral and cellular immune responses. Collectively, these findings provide novel insights for the development of efficacious broad-spectrum vaccines against ETEC infection.

## 2. Materials and Methods

**Figure 1:**
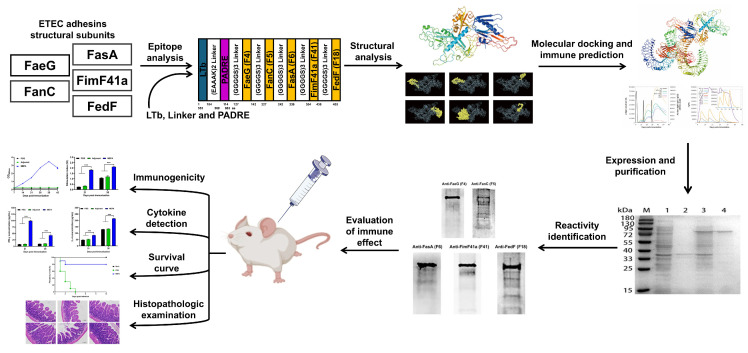
Schematic overview of the design, characterization, and evaluation of the ETEC MEFA vaccine. Data are presented as the mean ± SD from three independent experiments. *** *p* < 0.001. Scale bar = 100 µm in histopathologic examination.

The experimental design and overall workflow are shown in Figure 1.

### 2.1. Bacterial Strains and Plasmids

*Escherichia coli* isolates ETEC06 (O60:K88), ETEC1 (O101:K99:F41), ETEC3 (O20:987P) and ETEC5 (F18) were isolated and maintained at the Laboratory of Functional Microbiology and Animal Health of Henan University of Science and Technology. All *E*. *coli* were grown in LB medium at 37 °C. pCold I vector was used for protein expression.

### 2.2. Construction and Computational Modeling of Fimbria MEFA Proteins

To design the MEFA protein, physicochemical properties, signal peptides, transmembrane domains, phosphorylation sites, secondary structures, tertiary structures, and B cell epitopes of the FaeG, FanC, FasA, FimF41a, and FedF proteins were predicted and analyzed using ProtParam for physicochemical property analysis [22], TMHMM v.2.0 for transmembrane domain prediction [23], NetPhos 3.1 for phosphorylation site prediction [24], SignalP 5.0 for signal peptide prediction [25], PSIPRED for secondary structure analysis [26], AlphaFold3 for tertiary structure modeling [27], and the IEDB analysis resource for B cell epitope prediction [28]. Moreover, multiple segments of dominant B cell epitope sequences were subsequently screened. Furthermore, the dominant B cell epitopes from the five proteins were connected using flexible linkers, the universal T-helper (Th) cell epitopes PADRE and LT were incorporated, and their dominant epitopes were concatenated into the construct LTb-PADRE-K88-K99-987P-F41-F18.

### 2.3. Prediction of Secondary Structure

To further analyze the secondary structure of the MEFA protein, we employed the PSIPRED server, a publicly available online tool for protein structure prediction. By inputting the vaccine peptide’s amino acid sequence, PSIPRED generated detailed secondary structure predictions, providing critical insights into its folding patterns and conformational dynamics. These results enhance our understanding of the vaccine’s structural stability and potential functional implications [26].

### 2.4. Prediction, Refinement, and Quality Assessment of the 3D Structure of the MEFA Protein

To ensure greater accuracy in predicting the protein’s structure, the initial 3D model generated by AlphaFold3 was meticulously optimized using the GalaxyRefine system [29]. The refined model was then rigorously validated for structural integrity. First, its tertiary structure was assessed on the SWISS-MODEL server [30], followed by an evaluation of the overall protein architecture using the ProSA-web service [31].

### 2.5. Conformational Prediction of the B-Cell Epitope

B cell epitopes were identified from the refined 3D structure using ElliPro [32]. Average Protrusion Index (PI) scores were applied, with 0.9 as the threshold. ElliPro has demonstrated good accuracy in structural epitope prediction (AUC = 0.732).

### 2.6. Molecular Docking of MEFA Protein with Toll-like Receptors

The interaction of the MEFA vaccine with human TLR4 (PDB ID: 4G8A) was assessed using ClusPro [33]. Complexes were visualized in PyMOL, and interacting residues were mapped with PDBsum [34].

### 2.7. Immune Simulation Analysis

The immune response profile induced by the vaccine was simulated using C-ImmSim, an agent-based platform built on the Celada-Seiden model [35]. The vaccination protocol consisted of three administrations at 10 vaccine units per dose, with simulation steps set to 168. The first, second, and third injections were administered at steps 1, 42, and 84, corresponding to days post immunization 1, 14, and 28 of age, respectively.

### 2.8. Expression Purification and Western Blot Identification of MEFA

The LTb-PADRE-K88-K99-987P-F41-F18 construct was codon-optimized and cloned into the pCold I vector (Anhui General Biological Technology Co., Ltd., Chuzhou, China). The recombinant plasmid was first transformed into *E*. *coli* Top10 competent cells for propagation, then subsequently introduced into expression host *E*. *coli* BL21 (DE3) via heat-shock transformation. The recombinant strain was cultured overnight at 37 °C with shaking and then subcultured (1:100) into 5 mL ampicillin-containing LB medium. At OD_600_ ≈ 0.6, protein expression was induced with 0.2 mmol/L IPTG at 20 °C for 20 h. Cells were harvested, lysed by sonication, and centrifuged. The supernatant was purified using Ni-NTA affinity chromatography, with purified protein analyzed by SDS-PAGE.

The purified multiepitope peptide was analyzed using self-prepared polyclonal antibodies specific to K88, K99, 987P, F41, and F18 fimbriae (1:500), followed by HRP-conjugated secondary antibodies. Protein signals were detected using ECL substrate (Thermo Scientific, Rockford, IL, USA).

### 2.9. Animal Assay

Six-week-old female BALB/c mice were housed in accordance with the protocols approved by the Experimental Animal Center Institutional Committee of Henan University of Science and Technology. Mice were randomly allocated into three groups (*n* = 10/group)—the peptide group: multiepitope peptide vaccine (100 μg/dose) emulsified in Freund’s adjuvant; PBS group: sterile phosphate-buffered saline (PBS); and adjuvant group: Freund’s adjuvant alone (PBS emulsified in Freund’s adjuvant). All mice received three immunizations at 14-day intervals. Each dose (100 μL) was administered subcutaneously at distributed dorsal sites. Nine days after the third immunization, only the peptide group (*n* = 10) and the PBS control group (*n* = 10) were challenged via intraperitoneal injection with ETEC1 at a dose of 4 × LD_50_ (3.56 × 10^8^ CFU/mL) [36]. An additional age-matched unchallenged group (*n* = 10) was included as a baseline to monitor normal health and survival without ETEC infection, while the adjuvant-only group (*n* = 10) was not challenged and was included solely for immunogenicity assessments. All mouse groups were monitored daily for 10 days post challenge, with mortality rates recorded to calculate protection efficacy.

### 2.10. Determination of Serum Specific IgG Antibodies

Serum samples were obtained via retro-orbital bleeding at weekly intervals (days 7–42) post immunization. Longitudinal IgG antibody titers were quantified by indirect ELISA using ETEC multiepitope peptide-coated plates. Serum samples were serially diluted (1:2000–1:51,200) in PBS (100 μL/well), with parallel negative controls. Following 37 °C incubation (30 min), plates were treated with HRP-conjugated goat anti-mouse IgG (1:10,000) and developed with TMB substrate. Absorbance was measured at 450 nm using a microplate reader (Bio-Rad Laboratories, Inc., Hercules, CA, USA).

### 2.11. Lymphocyte Proliferation Experiment

Splenic lymphocytes were isolated from MEFA-immunized mice 7 days post secondary/tertiary immunization via density gradient centrifugation. Cells were adjusted to 7.55 × 10^6^ cells/mL in complete RPMI-1640 (10% FBS) and plated (50 μL/well) in 96-well plates. Three stimulation conditions were established—unstimulated control: 50 μL complete medium, positive control: 50 μL Con A (10 μg/mL, Sigma, St. Louis, MO, USA), and antigen-specific: 50 μL multiepitope peptide (10 μg/mL). Cells were cultured at 37 °C for 48 h under 5% CO_2_, and cell proliferation was assessed by a CCK-8 assay (10 μL/well, 4 h). The stimulation index (SI) was calculated as SI = (OD_450_ stimulated − OD_450_ blank)/(OD_450_ unstimulated − OD_450_ blank).

### 2.12. Determination of Specific IL-4 and IFN-γ Concentrations

Culture supernatants were collected from the unstimulated control, mitogen-stimulated, and antigen-stimulated splenic lymphocyte groups. Concentrations of murine IFN-γ and IL-4 in cell supernatants were quantified using commercial enzyme-linked immunosorbent assay (ELISA) kits according to manufacturer protocols (Beyotime Biotechnology, Shanghai, China).

### 2.13. Histopathological Study

After 24 h of challenge, the jejunums and ileums of each group of mice were collected. Following fixation in 13% neutral buffered formalin, tissue sections (5 µm) were prepared from paraffin-embedded blocks, stained with H&E, and assessed for pathological lesions using a microscope (Leica Microsystems, Wetzlar, Germany). Additionally, pathological scoring of the intestinal tissue sections was performed according to a six-tier standard [37].

### 2.14. Statistical Analysis

Statistical analyses were performed with GraphPad Prism 8.0 (GraphPad Software Inc., La Jolla, CA, USA). Group comparisons were evaluated using Student’s *t*-test or one-way ANOVA, with *p* < 0.05 considered statistically significant.

## 3. Results

### 3.1. Design and Computational Structure Analysis of MEFA

**Figure 2:**
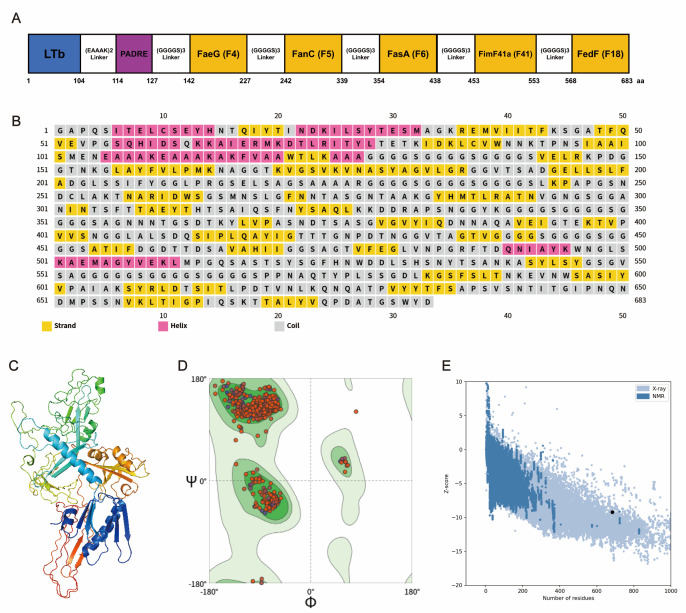
Secondary and three-dimensional structure prediction and quality assessment. (**A**) A graphical representation of the vaccine. (**B**) Predicted secondary structure of the vaccine sequence using the PSIPRED server. (**C**) Final 3D structure of the designed vaccine generated with AlphaFold3. (**D**) Ramachandran plot of the initial vaccine model. (**E**) Z-score plot evaluating the quality of the initial 3D structure.

The antigenic epitopes from the FaeG subunit of F4 fimbriae, the FanC subunit of F5 fimbriae, the FasA subunit of F6 fimbriae, the Fim41a subunit of F41 fimbriae, and the FedF subunit of F18 fimbriae were predicted using SOPMA and IEDB software (Table 1). The dominant epitopes of the above proteins with high surface accessibility, strong antigenicity, good flexibility, and hydrophilicity, located in β-turn or random coil regions, while devoid of α-helices and β-sheets, were further selected. The dominant epitopes from the five proteins were connected via flexible linkers, and the universal Th cell epitope PADRE along with the key LT epitope were incorporated to construct the chimeric antigen LTb-PADRE-K88-K99-987P-F41-F18 (Figure 2A).

The PSIPRED server was used to predict the secondary structure of the 683-amino-acid chimeric peptide, revealing a composition of 11.42% α-helices, 28.55% β-sheets, and a predominant 60.03% coils (Figure 2B). The secondary structure composition included a preponderance of coils in the vaccine’s structure, with smaller proportions of alpha helices and beta-sheets, which facilitates the exposure of antigenic recognition sites and enhances antibody binding accessibility. This structural arrangement is critical for the vaccine’s stability, solubility, and immunogenicity.

The three-dimensional (3D) structure of the designed vaccine was initially predicted using AlphaFold3 and subsequently refined with the GalaxyRefine server. Among the five generated models, the one with the highest Rama-favored score (98.5%) was selected for further analysis. The refined vaccine structure exhibited enhanced geometric quality, with 98.53% of residues in the Ramachandran-favored region, 0.29% outliers, and 0.4% in the rotamer region (Figure 2D). Structural validation metrics also improved, including MolProbity (1.11), Clash score (3.18), and ProSA Z-score (−9.2), indicating strong agreement with experimentally determined protein structures (Figure 2E). The final model was visualized using PyMOL (Figure 2C).

### 3.2. Prediction of B Cell Epitope Structure

**Figure 3:**
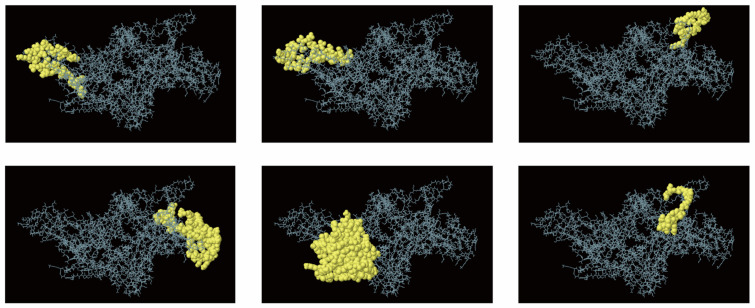
The three-dimensional (3D) structural B cell epitopes of the designed vaccine were predicted using the ElliPro tool. The gray sticks correspond to the vaccine construct, whereas the yellow surfaces depict the conformational B cell epitopes.

To identify conformational B cell epitopes, we employed ElliPro to analyze the multi-epitope vaccine, applying a threshold score of 0.7. The analysis yielded six discontinuous epitopes encompassing about 272 residues, with scores between 0.701 and 0.887. Each epitope contained 20–101 residues (Figure 3).

### 3.3. Molecular Docking Exploration

**Figure 4:**
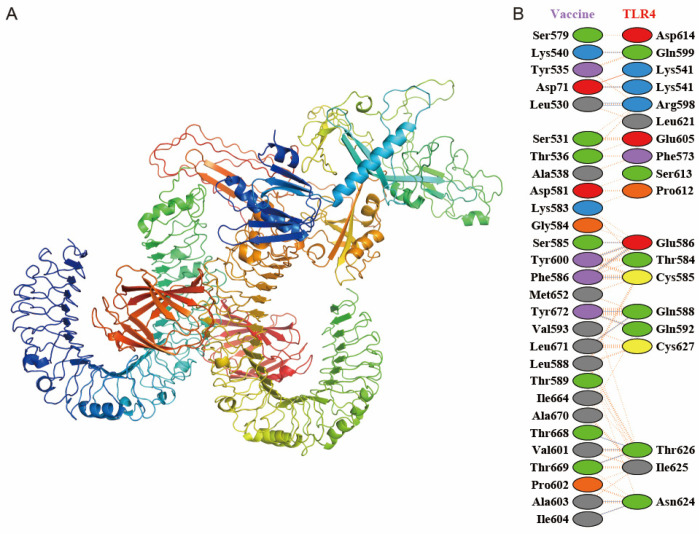
Characterization of the designed vaccine–TLR4 docking complex. (**A**) Overall view of the docked complex between the vaccine and TLR4. (**B**) Mapping of key residue interactions at the binding interface. The interaction types identified by PDBsum are indicated by color: salt bridges (red), disulfide bonds (yellow), hydrogen bonds (blue), and non-bonded contacts (orange).

Molecular docking of the vaccine construct with TLR4 was conducted using ClusPro 2.0 server. Among the 30 generated clusters per simulation, the lowest-energy conformation (ΔG = −828.3 kcal/mol) was identified as the most thermodynamically stable complex and subsequently visualized using PyMOL (Figure 4A). The vaccine–TLR4 interaction was analyzed for interfacial residues and binding strength using PDBsum. The results showed a complex with 28 interface residues (19 from TLR4) and an interface area of 1426 Å^2^ (Figure 4B). This interface featured one salt bridge, 16 hydrogen bonds, and 200 non-bonded contacts, which collectively demonstrate a high-affinity interaction.

### 3.4. Immune Response Simulation

**Figure 5:**
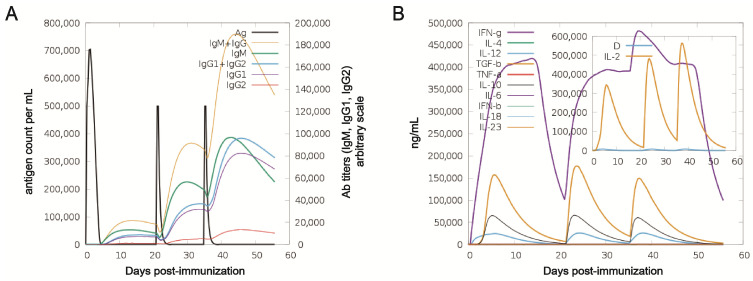
Immune simulation in silico. (**A**) Dynamics of antigen and antibody levels post immunization. (**B**) Concentrations of key cytokines and interleukins. The inset illustrates the corresponding danger signal (denoted as “D”).

Analysis of the simulation results showed a clear rise in antibody titers after successive immunizations, indicative of a robust humoral response (Figure 5A). Cytokine and interleukin levels also increased markedly after the second dose (Figure 5B). Together, these results suggest that the vaccine elicits high antibody production, demonstrating strong immunogenicity and potential efficacy against the target pathogen.

### 3.5. Expression, Purification and Western Blot Identification of MEFA

**Figure 6:**
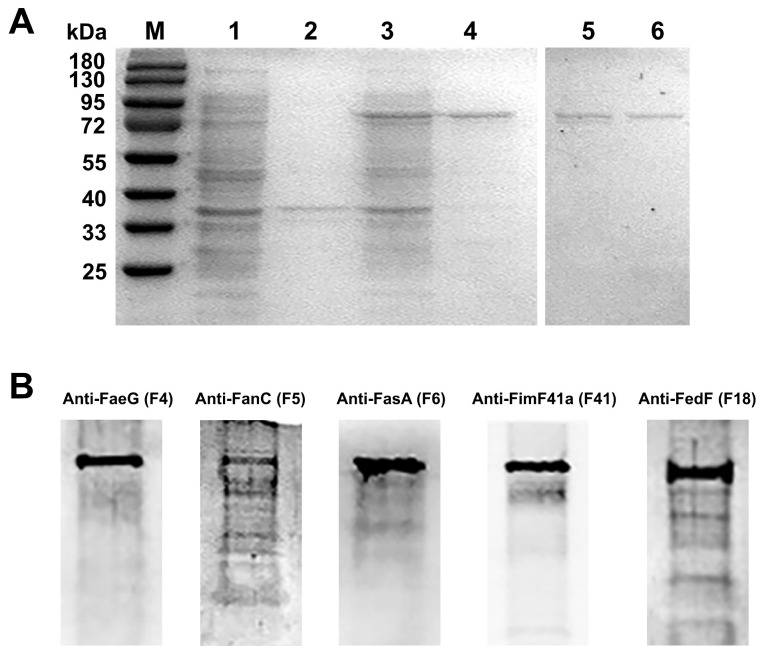
MEFA expression and reactogenicity. (**A**) Induction and expression of BL21-(pCold I -K88-K99-987P-F41-F18); M, protein marker; 1, supernatant of recombinant protein without induced; 2, precipitate of recombinant protein without induced; 3, supernatant of recombinant protein induced by IPTG; 4, precipitation of recombinant protein induced by IPTG; and 5–6, the purified MEFA protein. (**B**) The reactivity of different anti-adhesin structural subunit antibodies with MEFA protein were tested using Western blot. The original Western blot figures can be found in Supplemental Materials.

The target gene was modified, ligated into the pCold I vector, and transformed to obtain the recombinant polypeptide strain Top10-(pCold I-K88-K99-987P-F41-F18). MEFA proteins were expressed, extracted, and repurified using Nickel Affinity Chromatography. As demonstrated in Figure 6, the MEFA protein, with a molecular weight of approximately 76 kDa, demonstrates immunoreactivity with anti-FaeG, anti-FanC, anti-FasA, anti-Fim41a, and anti-FedF antisera (all used at a dilution of 1:500).

### 3.6. MEFA Protein Induced High Levels of Specific IgG Antibody in Mice

**Figure 7:**
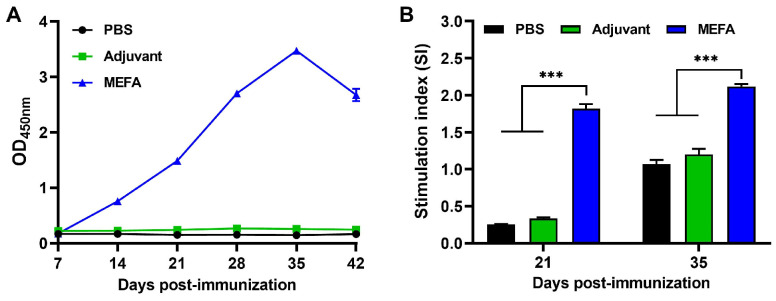
Immunogenicity of MEFA. (**A**) Dynamic level of specific IgG after immunization. MEFA effectively induced IgG antibody. (**B**) Lymphocyte proliferation experiment. Upon stimulation of splenic lymphocytes, splenocytes from MEFA-vaccinated groups exhibit significantly higher replication comparing with PBS group. Data are presented as the mean ± SD from three independent experiments. *** *p* < 0.001.

Mice were subcutaneously (s.c.) communized with the MEFA recombinant protein, and sera antibody titers were determined using the ELISA method, coated with the MEFA protein. The results showed that the MEFA-immunized group began producing specific antibodies at 7 days post primary immunization (7 dpi), followed by an exponential increase. The antibody titer peaked at 7 days post third immunization (35 dpi), reaching a high specific antibody titer of 1:256,000. In contrast, no specific antibody response was detected in either the PBS control group or the adjuvant-only control group. These above findings demonstrate that FaeG-FanC-FasA-FimF41a-FedF MEFA exhibits strong immunogenicity and effectively induces high-level humoral immune responses in vivo (Figure 7A).

### 3.7. MEFA Effectively Stimulated Proliferation of Murine Splenic Lymphocytes

The splenic lymphocytes in different immunized groups were stimulated with the MEFA protein for 48 h, followed by detection of lymphocyte proliferation using the CCK-8 assay kit. The results showed that the stimulation index (SI) values in the MEFA fusion protein-stimulated group at 7 days post second immunization (21 dpi) and 7 days post third immunization (35 dpi) were significantly higher than those in the control mice (*p* < 0.001). Moreover, the SI values of splenic lymphocytes in the MEFA polypeptide-stimulated group continued to increase with prolonged immunization time. The findings indicate that the FaeG-FanC-FasA-FimF41a-FedF fusion protein effectively stimulates proliferation of murine splenic lymphocytes, thereby inducing a robust cellular immune response (Figure 7B).

### 3.8. MEFA Stimulated the Production of Substantial Amounts of IFN-γ and IL-4 in Splenic Lymphocytes

**Figure 8:**
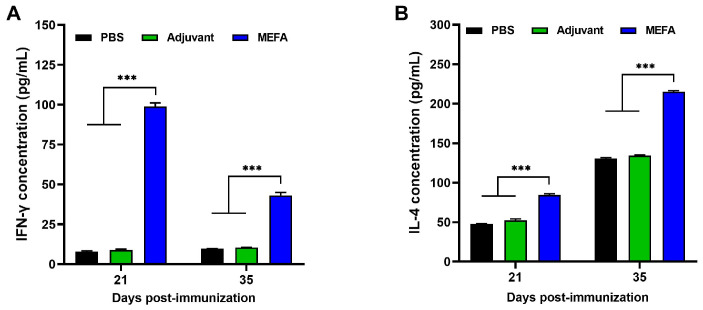
Determination of secreting-specific cytokines IL-4 and IFN-γ concentrations in splenocytes. Culture supernatants in spleen from unstimulated, mitogen-stimulated, and antigen-stimulated lymphocyte groups were analyzed for murine IFN-γ (**A**) and IL-4 (**B**) concentrations using commercial ELISA kits according to manufacturer’s protocols. Data are expressed as the mean and SD values from three independent experiments. *** *p* < 0.001.

IFN-γ and IL-4 levels in splenic lymphocyte supernatants were measured using a mouse cytokine ELISA kit. The results showed that the levels of IFN-γ and IL-4 in the supernatant of splenic lymphocytes from the MEFA-immunized group were significantly higher than those in the PBS and adjuvant groups (*p* < 0.001), and the cytokine concentrations increased over time (Figure 8A,B). The results further found that the MEFA fusion protein could stimulate the production of substantial amounts of IFN-γ and IL-4, with IL-4 levels being higher than IFN-γ, indicating that the immune response induced by the MEFA fusion protein was biased toward a Th2-type response.

### 3.9. MEFA Protein Conferred Protection Against Lethal ETEC Challenge in Mice

**Figure 9:**
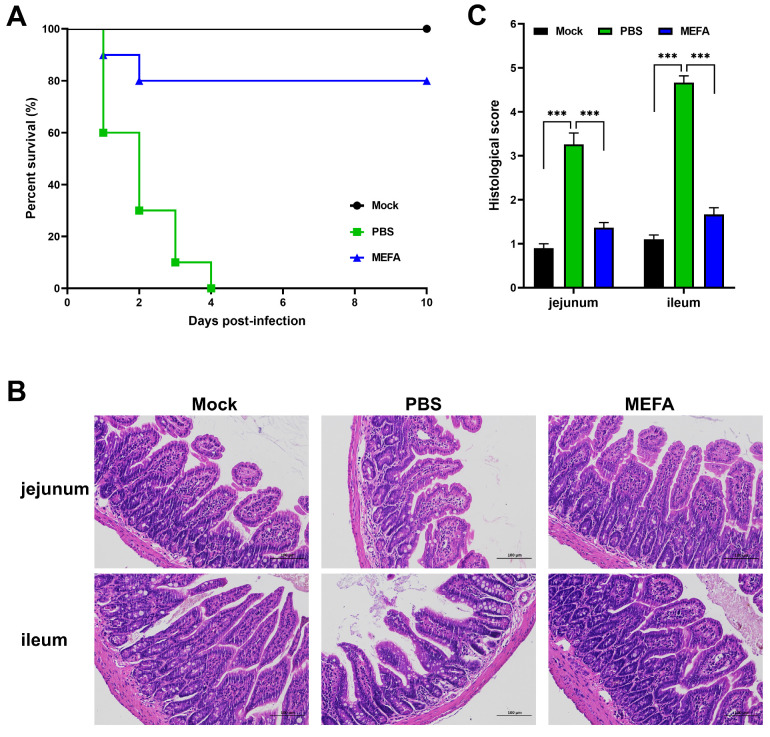
Assessment of MEAS immune protection by survival curve and histopathologic examination. (**A**) Post-challenge survival curve. Mice were randomly allocated into three groups (*n* = 10/group): a MEFA-immunized group, PBS group, and naïve mice group (mock). All mice were immunized three times at 14-day intervals. Following immunization, they were challenged with a lethal dose of ETEC1 (4 × LD_50_ per mouse) and monitored daily for 10 days. The protection rate of the vaccine was calculated based on survival outcomes. (**B**) Histopathological changes in the jejunum and ileum after challenge. At 24 h post challenge, jejunum and ileum samples were collected from each group, fixed in 13% neutral buffered formalin, and processed for histology. Paraffin-embedded 5 µm sections were H&E-stained and examined microscopically for lesions. (**C**) Pathological scoring of the intestinal tissue sections was performed according to a six-tier standard. Score 1, normal villi; Score 2, subepithelial stromal space and capillary congestion; Score 3, expansion of the submucosal stromal space with mucosal–submucosal separation; Score 4, progression of this separation to the lateral villous aspects; Score 5, villous blunting, exposed lamina propria vessels, and inflammatory cell infiltration; and Score 6, lamina propria disintegration, hemorrhage, or ulceration. *** *p* < 0.001.

The immunized groups were challenged with 4 × LD_50_ of ETEC1 to determine the protective efficacy. Within 10 days post challenge, all mice in the PBS group died, exhibiting diarrhea, perianal bedding adhesion, ruffled and dull fur, and lethargy before death. In contrast, mice in the MEFA-immunized group remained healthy with no obvious clinical symptoms. Furthermore, the protective rate was 80% in both MEFA-immunized groups (Figure 9A).

### 3.10. MEFA Protein Prevented Intestinal Tissue Damage Induced by Lethal ETEC

To further evaluate the protective effect of the FaeG-FanC-FasA-FimF41a-FedF MEFA immunization, jejunum and ileum samples were collected from mice in each group 24 h post challenge; the histopathological changes using HE staining were then examined. The results showed that PBS-challenged mice showed jejunal nuclear shrinkage, mucosal inflammation, ileal epithelial degeneration, and lymphoid infiltration compared to unchallenged controls. However, in the MEFA-immunized group post challenge, the jejunum exhibited well-distributed intestinal villi covered with a single layer of columnar epithelium, interspersed with goblet cells between epithelial cells, with a lower Chui’s score compared with the PBS group. The ileum also showed intact villous structures lined with a continuous monolayer of columnar epithelium, along with regularly arranged intestinal glands, and no significant pathological abnormalities were observed (Figure 9B,C).

## 4. Discussion

ETEC is one of the important pathogens causing diarrhea in domestic young livestock, which has caused huge losses in the breeding industry in recent years [38]. ETEC expresses various adhesins, with the major pathogenic ETEC strains prevalent in China and globally being those carrying the fimbrial adhesins K88 (F4), K99 (F5), 987P (F6), and F41 [39,40]. To date, with the intensification of antibiotic resistance, the development of ETEC vaccines against the five fimbrial adhesins has been urgently needed [3,41]. To overcome this challenge, this study demonstrates that a novel MEFA-based fusion protein (LTb-PADRE-K88-K99-987P-F41-F18) induces robust humoral and cellular immune responses, which confer comprehensive protection against ETEC challenge, including the prevention of intestinal tissue damage. Thus, this multivalent vaccine represents a promising new strategy for ETEC prevention in livestock farming operations.

The multiepitope fusion antigen (MEFA) vaccine exhibits advantages such as high efficacy, safety, and precision, but requires epitope analysis of antigenic proteins using computer software [42,43,44]. In this study, our analysis revealed that each of the five proteins possesses a transmembrane domain, which should be excluded during epitope selection. In terms of spatial conformation, α-helices and β-sheets are generally buried in the protein interior, hydrogen-bonded, and structurally rigid, thus limiting their recognition by antibodies. In contrast, β-turns and random coils typically form protruding, surface-exposed loops that are readily recognizable by antibodies [45,46]. Therefore, we selected the β-turns and random coils of the five proteins as backbone of MEFA protein. Based on the excellent agreement between the in silico predicted secondary and tertiary structures, the optimal antigenic epitopes are expected to exhibit surface accessibility, flexibility, and compatibility with antibody binding. Moreover, Western blot analysis confirmed the specific immune reactivity of the MEFA protein, as it was readily detected by antisera against FaeG, FanC, FasA, Fim41a, and FedF.

For the multi-peptide design in this study, flexible linkers were employed to link the epitopes from the five proteins, ensuring no unintended antigenic epitopes were introduced [47]. Additionally, to improve immunogenicity and circumvent MHC limitations, the Pan DR Epitope (PADRE)—a universal Th cell epitope—was integrated into the design, allowing the peptide to bind MHC-II (HLA-DP/DQ/DR) and strongly activate CD4+ T cells—with a potency up to 1000× greater than natural antigens [48]. Heat-labile enterotoxin (LT), an ETEC-derived hexameric protein, is both a key virulence factor and a crucial mucosal adjuvant [49]. Therefore, including LT’s immunodominant epitope in the MEFA design serves two purposes: expanding ETEC antigen coverage while leveraging LT’s adjuvant effects to boost immune responses. Immuno-informatics analysis showed stable vaccine–TLR4 binding interactions, demonstrating effective receptor binding and subsequent downstream signaling pathway activation. These findings were corroborated by immune simulation data, which showed a significant upregulation in antibody titers and elevated cytokine production, collectively indicating the induction of a potent adaptive immune response.

ETEC, a primary agent of animal diarrhea, colonizes the small intestine through adhesins and secretes enterotoxins during replication, causing diarrhea, dehydration, and emaciation. While oral gavage is standard for mimicking natural infection, we selected the intraperitoneal challenge—a model established in prior immunoprotection studies for its correlation with immunity and greater stringency due to its capacity to induce systemic infection—to rigorously evaluate our MEFA vaccine [50]. The results demonstrated that the MEFA candidate elicited robust antigen-specific immune responses and protected against lethal challenge, thereby offering significant mitigation of intestinal pathology in mice.

In summary, our study demonstrates that the MEFA protein is recognized by antibodies against the five major fimbriae subunits and induces robust IgG responses, lymphocyte proliferation, and splenic activation, indicating its potential as a multi-epitope vaccine candidate. Cytokine analysis further revealed a Th2-biased immune response, consistent with the observed humoral immunity. These results are in line with previous studies showing that fimbriae subunit or multi-epitope-based ETEC vaccines can elicit protective antibodies and reduce colonization in animal models [51,52]. Compared with these approaches, our MEFA construct integrates epitopes from five fimbriae into a single protein, potentially offering broader antigenic coverage. Similar multi-epitope strategies have been successfully applied to other pathogens, including Mycobacterium tuberculosis and influenza virus, highlighting the versatility of this approach [53,54]. Future studies should focus on evaluating bacterial clearance, challenges using natural infection routes, mucosal immune responses—particularly secretory IgA—and further optimization of epitope selection, adjuvants, and delivery systems to maximize vaccine efficacy and protective immunity.

## 5. Conclusions

In summary, the MEFA candidate vaccine developed in this study effectively elicited robust antigen-specific immune responses in mouse models. Crucially, it conferred significant protection against a lethal ETEC challenge and mitigated intestinal tissue damage, offering a preliminary experimental basis for future development of ETEC vaccines.

## Figures and Tables

**Table 1:** Dominant peptide segments of each protein.

Fimbrial Proteins	Dominant Epitopes	Selected Epitope Region
K88/FaeG	23-MTGDFNGSVD-32	84–198
	36-TITADDYRQK-45	
	61-LNDLTNGGTK-70	
	84-GRTKEAFATP-93	
	106-FTDYEGASVELRKPDGGTNK-125	
	134-PMKNAGGTKVGAVKVN-149	
	157-LGRGGVTSADGEL-168	
	184-LPRGSELSAGSAAAA-198	
K99/FanC	40-IEPEVNGNRT-49	70–166
	70-LKPAPGSNDC-79	
	103-SGNTAAKG-110	
	119-NVGNGSGGANIN-130	
	152-QLKKDDRAPSNGGYK-166	
987P/FasA	23-AAPAENNTSQA-33	97–180
	67-LKATGKGPAK-76	
	97-AGNNNTGSDTKYLVPASNDTSASG-120	
	163-GTTTGNPDTNGGVTAGTV-180	
F41/Fim41a	128-KNSGDNTEL-136	128–249
	154-DGDTTDS-160	
	208-MPGQSASTSYS-218	
	233-WDDLSHPNYTSADKASYLSYGSGVSAG-249	
F18/FedF	74 IPSSSGTLTCQAGT 87	74–262
	115 NESQWGQQSQ 124	
	151 AQTYPLSSGD 160	
	148 PPNAQTYPLSSGDLK 162	
	226 PNQNDMPSSN 235	
	251 YVQPDATGSWYD 262	

## Data Availability

The data generated and analyzed during this study are available from the corresponding authors on reasonable request.

## Supplementary Materials

The following supporting information can be downloaded at: https://www.mdpi.com/article/10.3390/vaccines13101057/s1, The original Western blot figures.
