# Supplementary figures and images for: Design and Evaluation of a Broadly Multivalent Adhesins-Based Multi-Epitope Fusion Antigen Vaccine Against Enterotoxigenic Escherichia coli Infection
Source: Vaccines (Basel). 2025 Oct 16;13(10):1057. doi: 10.3390/vaccines13101057 (PMC12567700; doi:10.3390/vaccines13101057)

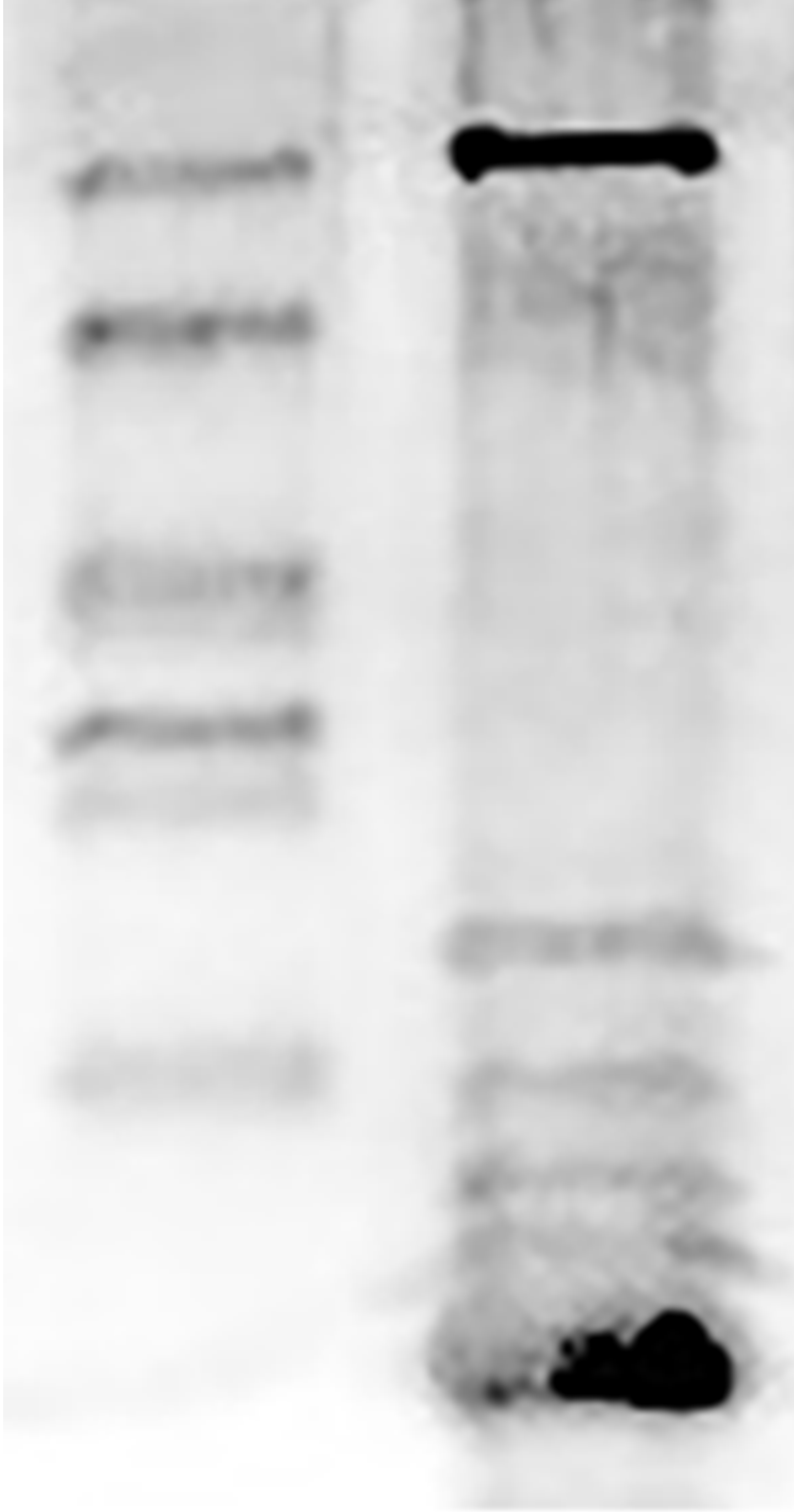

Supplement: Supplementary file 1 [file vaccines-13-01057-s001.zip › Figure 6B Anti-FaeG(F4) WB.tif]

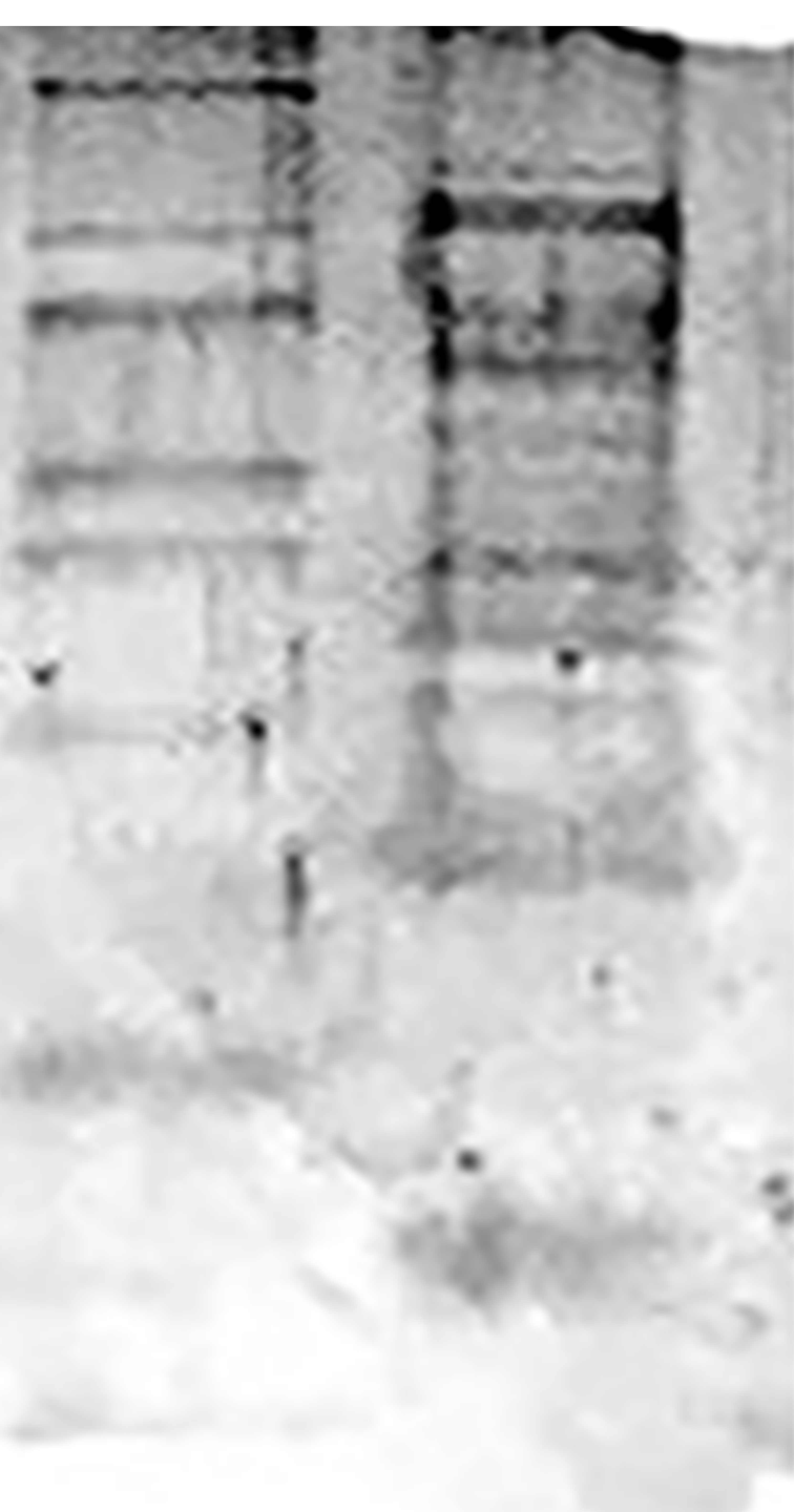

Supplement: Supplementary file 1 [file vaccines-13-01057-s001.zip › Figure 6B Anti-FanC(F5) WB.tif]

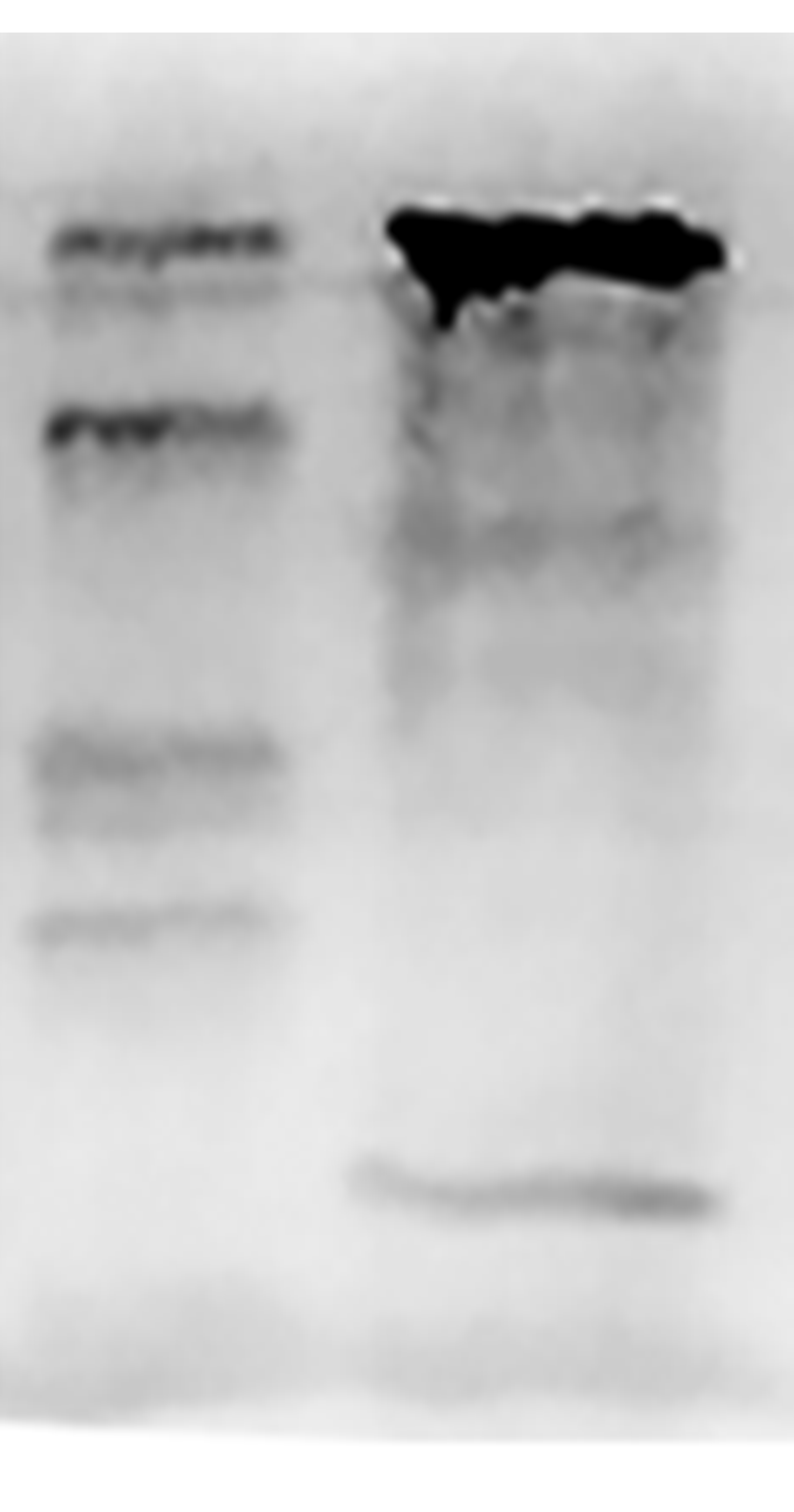

Supplement: Supplementary file 1 [file vaccines-13-01057-s001.zip › Figure 6B Anti-FasA(F6) WB .tif]

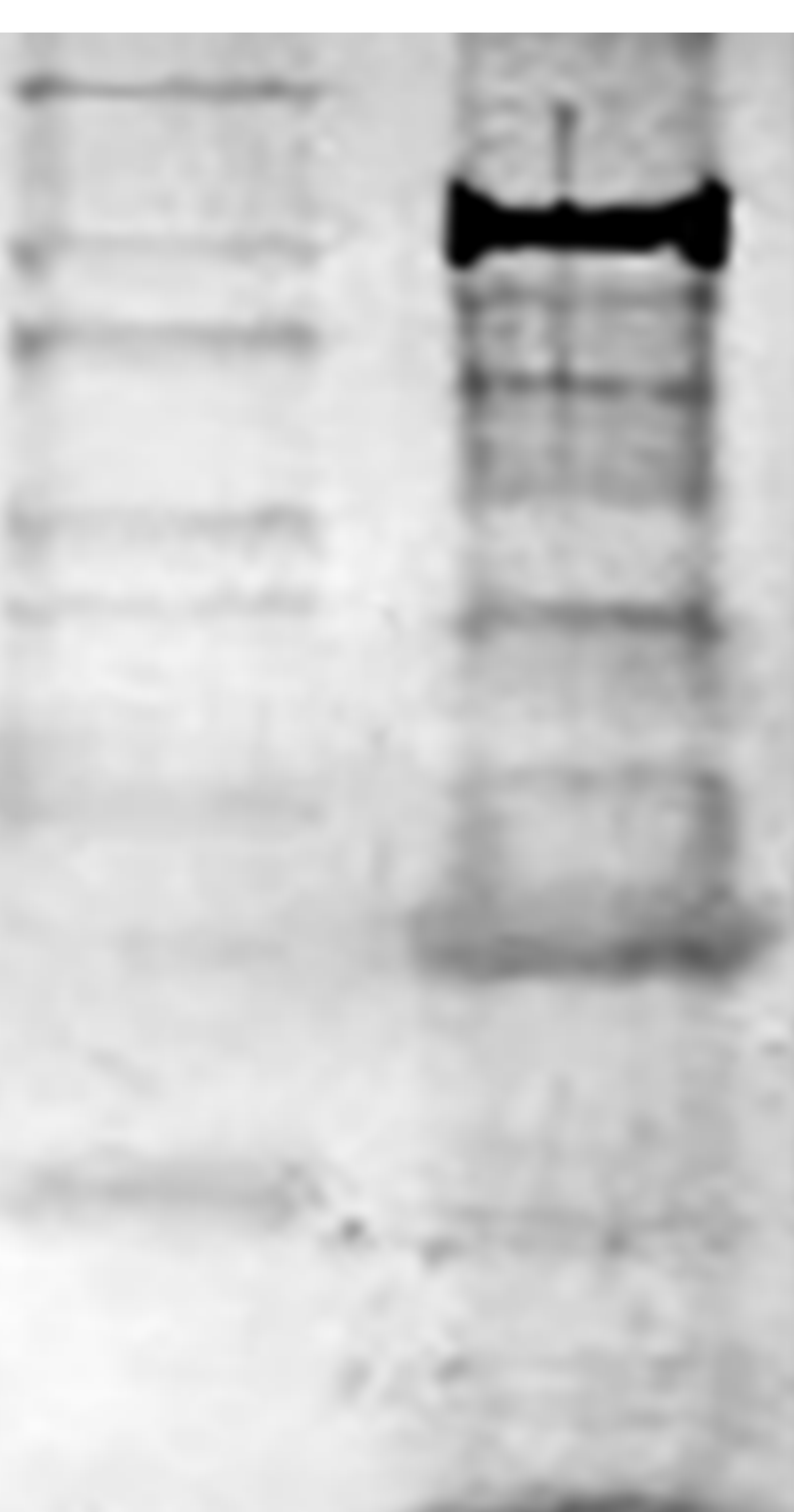

Supplement: Supplementary file 1 [file vaccines-13-01057-s001.zip › Figure 6B Anti-FedF(F18) WB.tif]

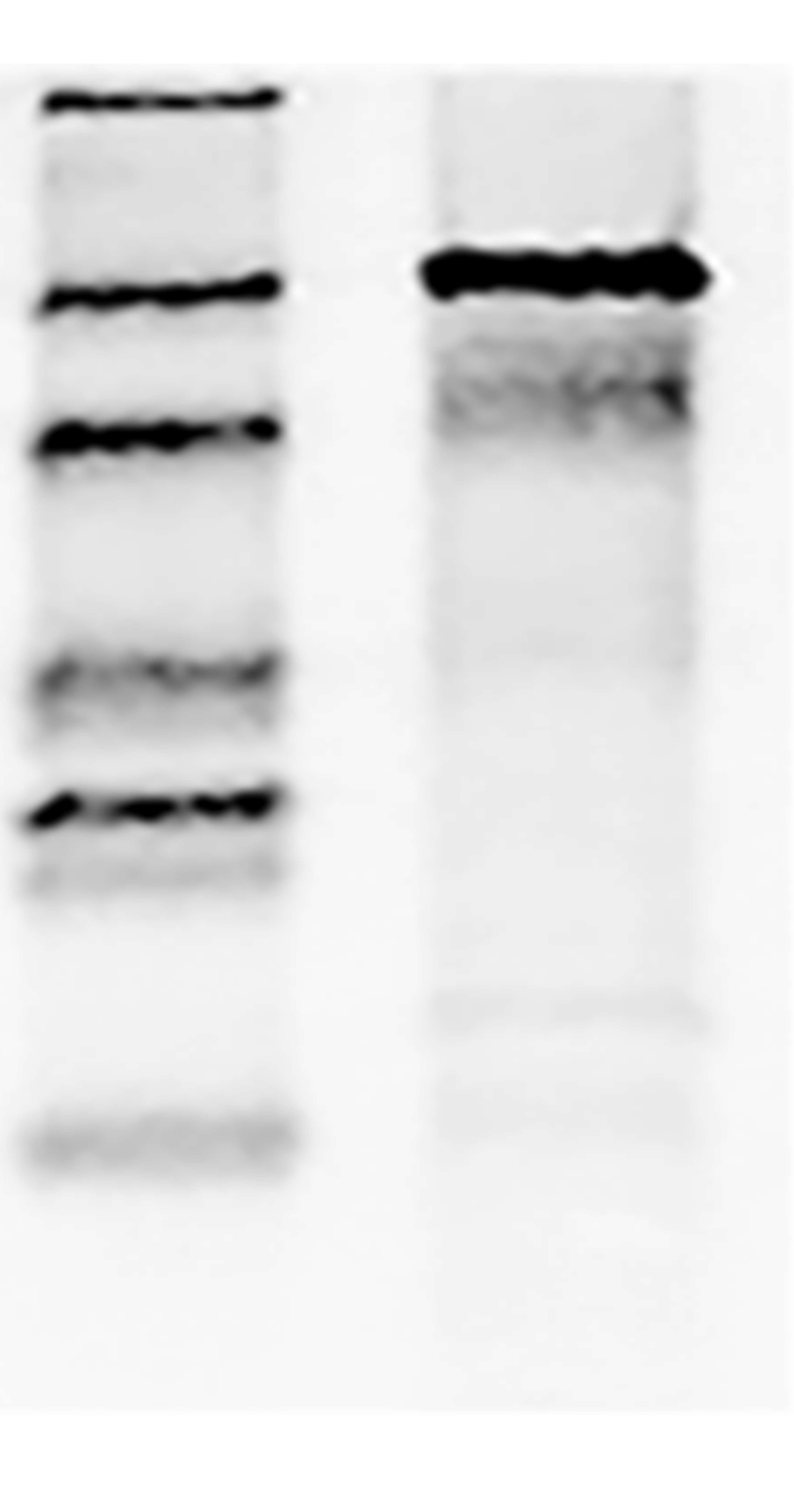

Supplement: Supplementary file 1 [file vaccines-13-01057-s001.zip › Figure 6B Anti-Fim41a(F41) WB.tif]
